# Clopidogrel Resistance Is Associated With DNA Methylation of Genes From Whole Blood of Humans

**DOI:** 10.3389/fgene.2020.583215

**Published:** 2021-01-15

**Authors:** Jin Yang, Qinglin Yu, Zhifeng Xu, Nan Zheng, Jinyan Zhong, Jiyi Li, Yahui Liu, Hongyu Xu, Jia Su, Lindan Ji, Xiaomin Chen

**Affiliations:** ^1^Department of Cardiology, Ningbo No. 1 Hospital, Ningbo, China; ^2^Department of Traditional Chinese Internal Medicine, Ningbo No. 1 Hospital, Ningbo, China; ^3^Department of Cardiology, Zhenhai People’s Hospital, Ningbo, China; ^4^Department of Cardiology, School of Medicine, Zhejiang University, Hangzhou, China; ^5^Department of Cardiology, Yuyao People’s Hospital of Zhejiang Province, Yuyao, China; ^6^Key Laboratory, Ningbo No. 1 Hospital, Ningbo, China; ^7^Department of Gerontology, Ningbo No. 1 Hospital, Ningbo, China; ^8^Department of Biochemistry, School of Medicine, Ningbo University, Ningbo, China

**Keywords:** DNA methylation, epigenomics, clopidogrel resistance, bioinformatics, coronary heart disease

## Abstract

Antiplatelet therapy has become a cornerstone in the treatment of coronary heart disease (CHD). However, due to high-residual-platelet-reactivity, clopidogrel resistance (CR) is a common phenomenon, and it is rarely known about the relationship between CR and epigenetic changes. This study compared the whole genomic methylation patterns of blood samples from patients with CR (*n* = 6) and non-CR (*n* = 6) with the Human Methylation 850K BeadChip assay. We explored differentially methylated CpG sites, genes, and pathways using bioinformatics profiling. The CR and control groups showed significantly different DNA methylation at 7,098 sites, with 979 sites showing hypermethylation and 6,119 sites showing hypomethylation. The pyrosequencing method was used to validate four differentially methylated CpG loci (cg23371584, cg15971518, cg04481923, cg22507406), confirming that DNA methylation was associated with the risk of CR (30 CR vs. 30 non-CR). The relative mRNA expression of the four genes (*BTG2*, *PRG2, VTRNA2-1*, *PER3*) corresponding to the loci above was also associated with CR, suggesting that alterations in DNA methylation may affect the expression of these four genes, eventually resulting in CR. Additionally, differentially methylated sites are partially related to genes and pathways that play key roles in process of circadian entrainment, insulin secretion, and so on. Hence, the mechanism and biological regulation of CR might be reflected through these epigenetic alterations, but future research will need to address the causal relationships.

## Introduction

The cause of mortality around the world is mostly from cardiovascular diseases, of which coronary heart disease (CHD) is the most fatal (Butler, 2011). For reduction of heart ischaemic events, the most commonly prescribed treatment for acute coronary syndrome (ACS) is antiplatelet agents, the most widely used of which are aspirin, clopidogrel, or a combination of both, which is also the groundwork for percutaneous coronary intervention (PCI) (Patrono et al., 2017). Unfortunately, because the responses of individuals to clopidogrel are significantly different, patients undergoing standard antiplatelet treatment still experience adverse cardiovascular events (Oliphant et al., 2016). Therefore, the safety and efficacy of platelet inhibition cannot be guaranteed in patients lacking a response to clopidogrel. Clopidogrel resistance (CR), which attenuates individual responses to clopidogrel therapy, is suggested to be the main reason for recurrent cardiovascular events. The prevalence is predicted to be approximately 30%, according to the European expert consensus guidelines in 2014 (Aradi et al., 2014).

The pathological mechanism of CR has been investigated in many analyses in recent years; clinical factors, drug-drug interactions and genetic factors have been assessed (Thomas and Storey, 2016; Warlo et al., 2019). Different clinical features, such as diabetes, gender, obesity, and hypercholesterolemia, contribute to a low clopidogrel response as well (D’ascenzo et al., 2014; Franchi and Angiolillo, 2015). Our previous study showed that male sex, high albumin, and reduced hsCRP are risk factors for CR in a Chinese Han population (Su et al., 2017). Drug interactions can also lead to differences in the clopidogrel response. It was reported that combining drugs such as ketoconazole, proton pump inhibitors (PPIs), and statins with clopidogrel might increase the risk of CR (Chen et al., 2013; Park et al., 2017). In genetics, research on CR is currently focused on genetic polymorphisms. Clopidogrel is a thienopyridine prodrug, so hepatic activation is essential for the antiplatelet effect (Zhang et al., 2017). The single nucleotide polymorphisms (SNPs) of cytochrome P450 isoenzymes (CYPs) in the liver are associated with the success or failure of this metabolic process (Siller-Matula et al., 2013). The main mutant alleles, CYP2C19^∗^2 (frequency of 30–50%) and CYP2C19^∗^3 (frequency of 5–10%), are the most common genotypes in Asian patients (Xie et al., 2011). The SNPs of other drug-metabolizing enzymes (CYP2C9, CYP3A4, CYP2B6, and CES1), transporters [ABCB1 (ATP-binding cassette subfamily B member 1)], and action receptors (P2Y12) have also been researched (Tarkiainen et al., 2015; Thomas and Storey, 2016; Pan et al., 2017). However, the mechanisms of CR have not been fully elucidated, and the known genetic variants could only explain a small portion of the variability.

Recently, research on pathological mechanisms has focused on epigenetics, which may be responsible for many diseases, such as congenital heart disease (Lyu et al., 2018). Epigenetic alterations are mainly composed of DNA methylation, histone modification, and non-coding RNAs (lncRNAs and miRNAs). DNA methylation, another important component of epigenetics, may significantly influence the clopidogrel response. Changes in the blood-derived DNA methylation in some diseases, such as myocardial infarction and CHD, have been observed (Ghaznavi et al., 2018). We found the effect of DNA methylation in the ABCB1 (Su et al., 2017), P2Y12 (Su et al., 2014), and PON1 (paraoxonase 1) genes (Su et al., 2019) on CR. However, it is rarely known about DNA methylation alterations in the peripheral blood of subjects with CR. Alterations in DNA methylation modification could influence the expression and function of genes, leading to the diversity of the antiplatelet responses of clopidogrel. Additionally, the signaling pathways involved in regulation are poorly understood, which will be further and fully explored in our study.

Thus, the present study on patterns of blood whole-genome methylation was performed among ACS objects who underwent PCI and had diverse residual platelet reactivity to clopidogrel, identifying various methylation sites. Pathway enrichment analysis was performed on genes with differential methylation sites to find possible signaling pathways for the low response to clopidogrel.

## Materials and Methods

### Study Population

From January to October 2018, a total of 36 patients with CR and 36 non-CR controls were enrolled in this study; all subjects were all Han Chinese in Ningbo selected from Ningbo First Hospital. All patients and controls were ACS patients who underwent PCI using drug-eluting stents and were older than 18 years. The case group met the diagnostic criteria for CR: clinical confirmation of clopidogrel antiplatelet therapy failure and reaction units of P2Y12 (PRU) measured by the VerifyNow P2Y12 assay (Accumetrics, Inc., San Diego, California) greater than 240 (Marcucci et al., 2009). The patients and the controls did not have hepatic or kidney function disorders, rheumatoid-related diseases, severe infection, or a history of active bleeding. All blood samples and clinical data were obtained after informed consent was provided by the patients or their guardians. The study protocol was implemented under appropriate ethical procedures supported by the Ethics Committee of Ningbo First Hospital, which complied with the Declaration of Helsinki.

### Genomic DNA Extraction

Peripheral blood samples from 36 subjects with CR and equal numbers of non-CR subjects were collected, 12 of which were used for the global DNA methylation analysis (these six CR and six non-CR patients were gender matched), and the remaining samples (30 CR and 30 non-CR) were used for validating the DNA microarray results. Blood samples were stored frozen in a -80°C freezer.

The purified DNA from human whole blood cells was extracted with a QIAamp DNA Blood Mini Kit (Qiagen, Hilden, Germany) after the frozen blood samples were thawed. DNA concentration and purity were quantified in a NanoDrop 2000 system (NanoDrop, Wilmington, DE) by measuring the optic density ratio of the maximal absorbent wavelengths from 260 to 280 nm (OD260/280). The DNA concentration must be higher than 50 ng/μl, and the OD260/280 ratio must be from 1.60 to 2.10.

Each qualified DNA sample was transferred to a centrifuge tube according to the kit instructions and then placed in a −20°C refrigerator. Agarose gel electrophoresis with ethidium bromide staining was used to determine the extracted DNA integrity.

### 850 BeadChip DNA Methylation Assay

Whole DNA methylation (six CR vs. six non-CR) was assessed with the Infinium Human Methylation 850 BeadChip Kit (Illumina, Inc., San Diego, CA, United States), which covers the human genome’s 853,307 cytosine positions. A series of steps were taken for the processing of DNA samples in accordance with the instructions. DNA samples first underwent DNA denaturation, followed by whole genome amplification, fragmentation, precipitation and resuspension, and hybridization to arrays.

### Validation of Differentially Methylated CpG Loci (DML)

When the status of the differential methylation sites was confirmed, 4 CpG sites with different degrees of methylation (cg04481923, cg15971518, cg23371584, cg22507406) were selected for cross-validation in blood DNA samples from 30 CR cases and 30 controls, which was in an independent cohort. Thus, each blood DNA sample in both groups was double-vulcanized based on the manufacturer’s instructions. We used the PyroMark PCR kit (Qiagen, California, United States) to perform PCR amplification on the region of interest. Nucleotide probes included a biotinylated version allowing detection by streptavidin Sepharose (Table 1). We purified and further processed the single-stranded DNA that had been biotinylated.

**TABLE 1:** Primers for cg04481923, cg15971518, cg23371584, and cg22507406 in analysis of the CpG island loci.

	**Group**	**DNA sequence (5**′**→3**′)	**Modification**	**Purification**
cg04481923	Forward primer	AATTTATTGTATAAAAGGGTTAGTAAGTAT		PAGE
	Reverse primer	CATACCCAACTTTCTATCTATCCATCTCTA	5′Biotin	HPLC
	Sequencing primer	CTATCCATCTCTATACTAAAATTC		PAGE
cg15971518	Forward primer	GTTGGTTAATTGAGTGGTTTGATTTAGAA	5′Biotin	PAGE
	Reverse primer	AAAACTTCCCAACTAACTATAATAAAAAA		HPLC
	Sequencing primer	AGAAGGAAAGAAGTTAGATA		PAGE
cg23371584	Forward primer	AATTTTATGGAAGTTGGAAAGATTTATT	5′Biotin	PAGE
	Reverse primer	ATACCAAAAACAATACACTAACCACTAAA		HPLC
	Sequencing primer	CTAAAATCCCCTCCCA		PAGE
cg22507406	Forward primer	TAGGTTATTTGGAAAAGGTATTTAATTAGG		PAGE
	Reverse primer	AACCAAACTAATCTTAAACTCCTAAC	5′Biotin	HPLC
	Sequencing primer	ATTTAATTAGGTGAAGGAGAAAATA		PAGE

We validated the relative mRNA expression of four genes (BTG2, PRG2, VTRNA2-1, PER3) corresponding to the four loci above through qRT-PCR. An RNeasy Plus Universal Kit (Qiagen) was used to extract the RNA. The PrimeScript^TM^ RT Reagent Kit was used to synthesize cDNA with gDNA Eraser (TaKaRa Bio, Kusatsu, Japan), and 1 μg RNA was applied. Template cDNAs were diluted 1:4, and an ABI 7500 Quantitative Real-time PCR (qRT-PCR) System (Applied Biosystems, Foster City, CA) was used to quantify the relative expression of the four genes. The housekeeping gene GAPDH was used for normalization. Primer Premier 5 was used to design the primers (Table 2) for qRT-PCR amplification. After the sample was measured in triplicate, the average value was taken. The mRNA levels of the four genes were calculated with the relative quantitative method.

**TABLE 2:** qRT-PCR primers for *BTG2*, *PRG2*, *VTRNA2-1*, and *PER3* for gene analysis.

	**Group**	**DNA sequence (5’→3’)**
*BTG2*	F	CTGGAACGGTGAAGGTGACA
	R	AAGGGACTTCCTGTAACAATGCA
*VTRNA2-1*	F	AAGGGTCAGTAACCACCCGCG
	R	CGGGTCGGAGTTAGCTCAAGCGG
*PER3*	F	TGGTGGTGGTGAATGTAAGAC
	R	GGCTGTGCTCATCGTTCC
*PRG2*	F	CTCCACCTCCCTGCAATAAA
	R	AGGTGCCCAGTAATCAGGTG

### Statistical Analysis

We used SPSS 21.0 to perform statistical analysis. The distribution of variables was tested with the Kolmogorov-Smirnov normal distribution test. Means of designated comparison groups were compared by Student’s *t*-test or nonparametric test. Logistic regression was utilized for analyzing whether confounding variables influenced on CR. A 2-sided *P* < 0.05 was regarded as the minimal statistically significant level of difference.

The β-values were calculated from the obtained methylation data and the signal of the final hybridization products. The average β-value indicates the methylation levels for each given site, ranging from 0 to 1 (0 indicates not methylated, 1 indicates completely methylated). The calculation formula for the β-value is as follows: β = max (signal B, 0)/[max (signal A, 0)+max (signal B, 0)+100]. After the methylation assay, the data were processed in the following order: background correction, probe scaling, quantile normalization, and logit transformation. We performed differential methylation analysis based on logit-transformed values and conducted the Wilcox rank test to compare six CR samples to six non-CR samples. The Benjamini–Hochberg method was used to correct *P*-values by calculating the false discovery rate. Probes with adjusted *P* < 0.01, FDR < 0.05 and delta β 0.2 or < -0.2 were considered differentially methylated and statistically significant.

GraphPad Prism 7 was used to assemble the figure panels. The BeadStudio Methylation Module v3.2 software (Illumina, Inc.) (http://www.illumina.com) was used to select and analyze differential DNA methylation sites. PyroMark Q24 software (Qiagen, CA, United States) was used to analyze the pyrosequencing data. The MultiExperiment Viewer (MeV) was utilized to perform microarray clustering analysis. The Database for Annotation, Visualization and Integrated Discovery (DAVID) bioinformatics database (http://david.abcc.ncifcrf.gov/home.jsp/) was used to select differentially methylated genes. The Gene Ontology (GO) (http://geneontology.org/) and Kyoto Encyclopedia of Genes and Genomes (KEGG) (http://www.genome.jp/kegg/) databases were used for ontological profiling and genomic network profiling of the differentially methylated genes.

## Results

### Characteristics of the Patients Included in the Microarray Analysis

Total DNA from the peripheral blood samples of six patients and six controls was extracted for the blood genomic DNA methylation assay. The clinical characteristics of these 12 patients (six CR and six non-CR) are presented in Table 3. The clinical characteristics were matched, and no significant differences were observed between these two groups.

**TABLE 3:** Characteristics of the patients included in the microarray analysis.

**Index**	**CR (*n* = 6)**	**Non-CR (*n* = 6)**	**t/z**	***P*-value**
Gender (male/female)	3/3	3/3	–	–
Age (years)	66.0 ± 7.0	59.5 ± 9.0	–1.403	0.191
Weight (kg)	60.8 ± 9.7	64.0 ± 10.0	0.556	0.590
MAP (mmHg)	99.1 ± 12.6	85.6 ± 15.0	–1.694	0.121
BMI (kg/m^2^)	22.6 ± 4.1	24.1 ± 3.1	0.705	0.497
Total cholesterol (mmol/L)	4.44 ± 0.64	5.54 ± 1.53	1.624	0.150
Blood sugar (mmol/L)	6.643 ± 2.839	6.630 ± 2.58	–0.009	0.993
Triglyceride (mmol/L)	2.058 ± 0.791	3.128 ± 2.700	0.931	0.388
Albumin (g/L)	41.67 ± 4.63	39.78 ± 5.63	–0.633	0.541
HDL (mmol/L)	1.023 ± 0.252	0.922 ± 0.186	–0.794	0.445
LDL (mmol/L)	2.413 ± 0.690	3.200 ± 0.763	1.837	0.091
HbA1c (%)	6.88 ± 1.84	6.83 ± 1.55	–0.051	0.960
LVEF (%)	60.7 ± 8.0	67.2 ± 3.8	1.789	0.116
PLT (10^9^/L)	180.5 ± 61.7	262.7 ± 140.3	1.313	0.219
MPV (fl)	8.80 ± 1.82	7.92 ± 1.21	–0.989	0.346
PDW (fl)	16.78 ± 0.80	16.57 ± 0.52	–0.554	0.591
ALT (U/L)	35.7 ± 28.6	40.7 ± 22.1	0.339	0.742
AST (U/L)	149.8 ± 193.0	163.3 ± 141.2	0.138	0.893
BUN (mmol/L)	5.333 ± 0.806	5.995 ± 0.918	1.327	0.214
Uric acid (μmmol/L)	282.2 ± 133.7	260.3 ± 80.9	–0.342	0.739
Cr (μmol/L)	65.75 ± 21.46	57.72 ± 14.58	–0.758	0.466
PCT (μg/L)	0.1 ± 0.03	0.2 ± 0.09	1.256	0.238
HsCRP (mg/dL)	2.330 (1.098, 3.803)	9.405 (1.398, 38.173)	–0.961	0.394

### Global Changes in Blood Genomic Methylation Patterns in CR

**FIGURE 1:**
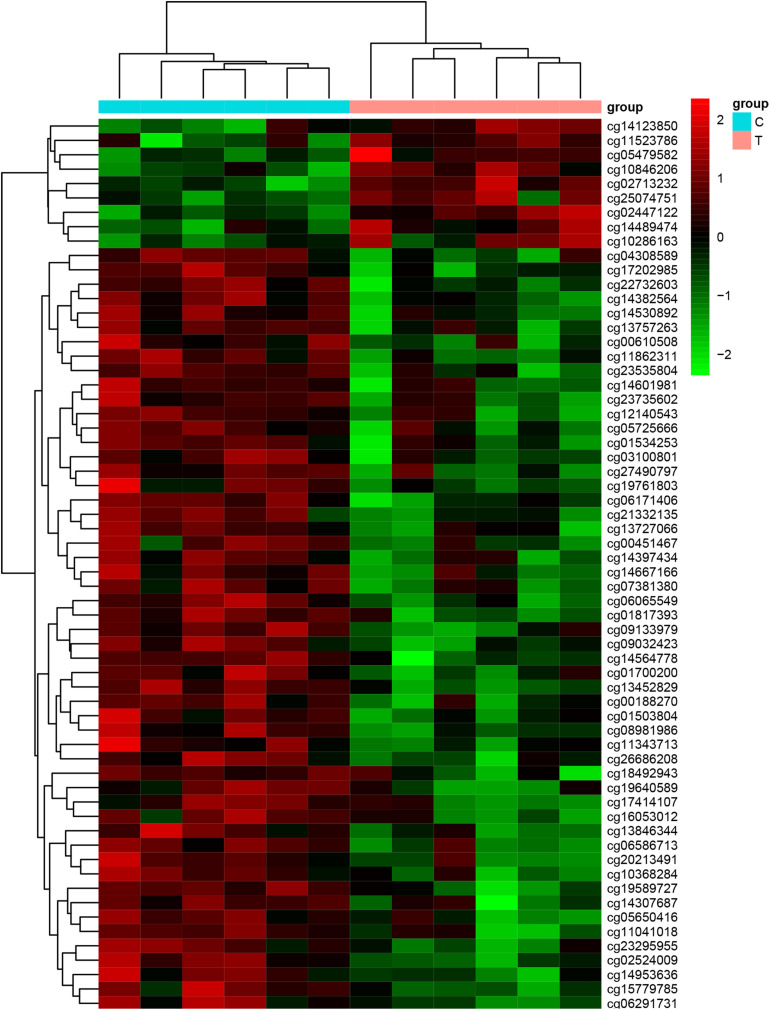
Heat map generated from clustering analysis indicating differentially methylated DNA sites of the whole blood genes of the control (*n* = 6) and CR (*n* = 6) groups (C, the patients with non-CR; T, the patients with CR).

**FIGURE 2:**
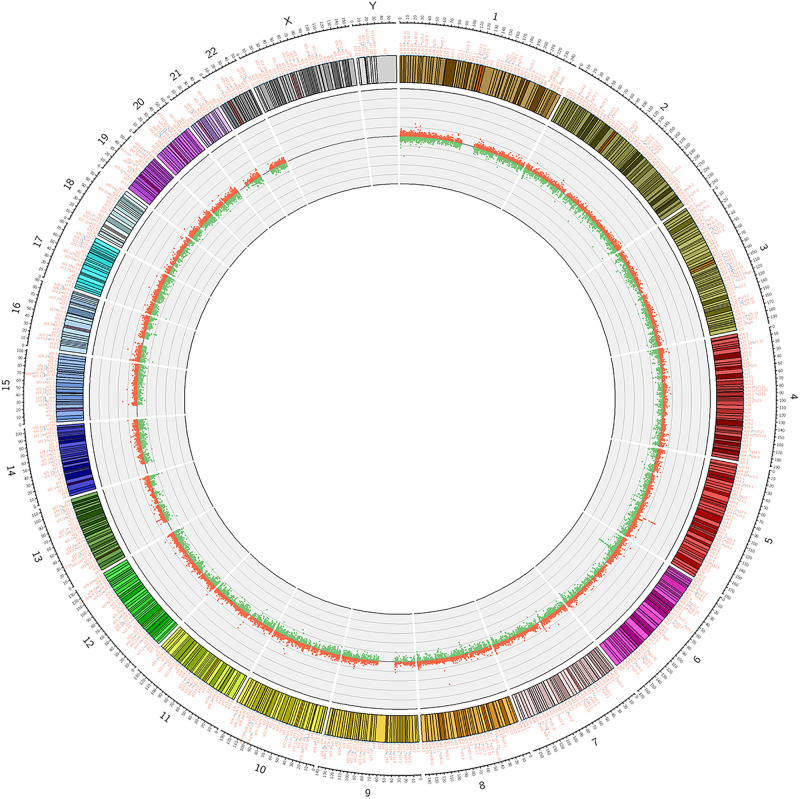
Circosplot for the whole genomic methylation patterns of the non-CR (*n* = 6) and CR (*n* = 6) patients. The hyper- and hypomethylation sites in 23 chromosomes (red dots: hypermethylation, green dots: hypomethylation).

We used Genome Studio V2011 software to report the β-values of 853,307 DNA methylation sites for the samples from the controls (*n* = 6) and the patients with CR (*n* = 6) (Figure 1). Statistical analysis revealed that 7,098 sites in all showed a difference in the degree of methylation. 979 sites were hypermethylation and 6,119 sites involved hypomethylated among them, with a ratio of 0.16. Then, we plotted the distribution of differentially hypo- and hypermethylated sites across chromosomes (Table 4 and Figure 2). Based on the result, there were the most hypomethylated sites on chromosomes 1, 7, and 11, the most hypermethylated sites on chromosomes 1, 2, and 3, and the most overall differentially methylated sites on chromosomes 1, 2, and 11.

**TABLE 4:** Frequency of differentially methylated sites by chromosome after normalization to chromosome length.

**Chromosome**	**Frequency hypo**	**Frequency hyper**	**Total frequency**
1	575	84	659
2	475	75	550
3	341	66	407
4	268	44	312
5	349	61	410
6	345	64	409
7	366	44	410
8	270	36	306
9	221	20	241
10	308	52	360
11	378	63	441
12	291	66	357
13	152	18	170
14	218	42	260
15	163	37	200
16	288	36	324
17	280	57	337
18	112	16	128
19	264	47	311
20	200	31	231
21	88	10	98
22	167	10	177

**FIGURE 3:**
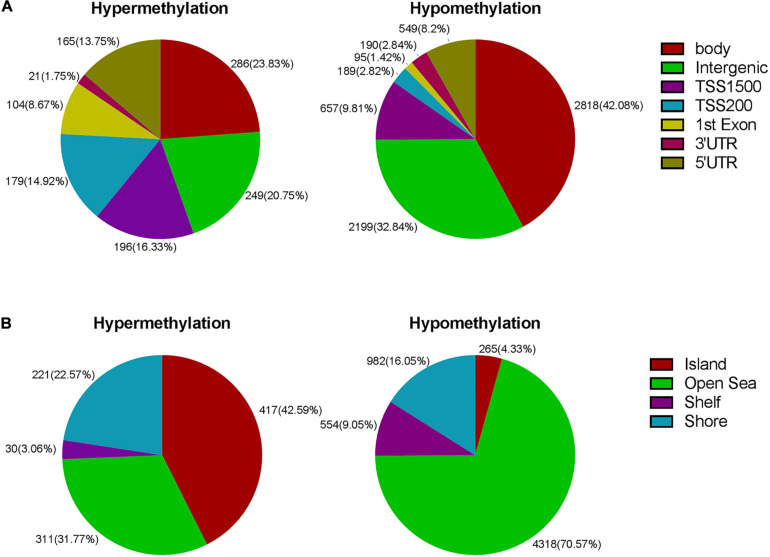
**(A)** Frequency of differentially methylated regions according to functional distribution. **(B)** Frequency of differentially methylated sites according to CpG island contextual distribution.

Next, we carried out analysis according to functional domains of DNA. 286 sites were located at the gene bodies (23.83%) among the hypermethylated sites, followed by 249 sites at noncoding intergenic domains (20.75%) and 196 (16.33%) sites situated within 1,500 bp upstream of the TSS (Figure 3A). Among the hypomethylated sites, there were 2,818 sites located at gene bodies (42.08%), followed by 2,199 sites at noncoding intergenic domains (32.84%), and 657 sites situated within 1,500 bp upstream of TSS (9.81%) (Figure 3A). Both mostly hypermethylated sites and mostly hypomethylated sites were located in the body regions. In addition, the smallest percentage (1.75%) was located in the 3’UTR for hypermethylated loci, while mostly hypomethylated loci had the smallest percentage in the 1st exon (1.42%) (Figure 3A). Among the 7,098 differentially methylated sites, 2,134 sites was located at promoter regions, the ratio (644/1,490 = 0.43) of hyper- to hypomethylated sites was higher than which (979/6,119 = 0.16) in the whole genome.

The regional distribution of the differentially methylated CpG loci was assessed according to their distance from the nearest CpG island. An and B are away from location C, 2 and 3 km, respectively. The regions of shores and shelves are away from CpG island 0–2 and 2–4 kb, respectively, and the regions of open sea are isolated loci. When comparing the CR group to the control group, we observed that most of hypermethylated loci (42.59%) were found in CpG islands, while most of hypomethylated loci were located in the open sea (70.57%) (Figure 3B). The smallest percentage (4.33%) of hypomethylated loci was located in islands, compared to the smallest percentage of hypermethylated sites (3.06%) found in shores (Figure 3B).

### Relevance of Differentially Methylated DNA Sites to Functional Genes

With the DAVID bioinformatics database, the differentially methylated sites were analyzed in relation to known functional genes. Among the 7,098 differentially methylated sites, the top ten genes with the greatest extent of hypermethylation were LILRA3, BTG2, PITX1, NDRG4, ARNT2, ESPNL, SPEG, SULT1A1, PTPN21, and SDC2. Three of the above 10 genes’ relevant differentially methylated DNA sites were situated in the region of body, as well as in TSS1500 and TSS200. A subset of genes of either the hypermethylated or hypomethylated genes had been shown to have single nucleotide polymorphisms (SNPs) from the query sites (Table 5). The top ten genes with the greatest extent of hypomethylation were VTRNA2-1, VTRNA2-1, VTRNA2-1, VTRNA2-1, FRG1B, VTRNA2-1, VTRNA2-1, VTRNA2-1, and PRG2. Among this latter set, 8 of 10 hypomethylated sites were associated with SNPs, while eight sites were located around the promoter region and two in the gene body (Table 5).

**TABLE 5:** Top 10 hyper- and hypomethylated genes in the whole blood genome in CR.

	**Target ID**	**Gene ID**	**Gene name**	**Delta β**	***P*-value**	**CHR**	**Location**	**SNP**
Top 10 hypermethylated genes	cg21898358	LILRA3	Leukocyte immunoglobulin like receptor A3	0.317164492	0.00679633	19	TSS1500	rs574131286; rs538649487
	cg23371584	BTG2	BTG anti-proliferation factor 2	0.233891104	0.005231472	1	Body	rs190670739; rs10920608
	cg25648267	PITX1	Paired like homeodomain 1	0.088083459	0.009003961	5	TSS1500	rs180702771; rs58496773
	cg00758881	NDRG4	NDRG family member 4	0.20813473	0.00481791	16	Body	rs559344283
	cg07935500	ARNT2	Aryl hydrocarbon receptor nuclear translocator 2	0.045366334	0.009557636	15	TSS200	rs569641600
	cg06574296	ESPNL	Espin like	0.298913067	0.007608362	2	TSS1500	rs557989371; rs200123963; rs115142731
	cg01727145	SPEG	Striated muscle enriched protein kinase	0.057670765	0.006838814	2	Body	rs541579349
	cg17281975	SULT1A1	Sulfotransferase family 1A member 1	0.031473562	0.005910516	16	TSS200	rs536626026; rs191470441; rs570036940
	cg13037201	PTPN21	Protein tyrosine phosphatase non-receptor type 21	0.118722943	0.008966412	14	5’UTR	rs577225064
	cg13096260	SDC2	Syndecan 2	0.059431928	0.002173929	8	TSS200	rs143598052
Top 10 hypomethylated genes	cg04481923	VTRNA2-1	Vault RNA 2-1	-0.43410451	0.000155542	5	Body	rs538710441; rs553551499; rs572325590
	cg08745965	VTRNA2-1	Vault RNA 2-1	-0.31600589	0.001333554	5	TSS1500	rs71589303; rs9327740
	cg18678645	VTRNA2-1	Vault RNA 2-1	-0.3511922	0.002162832	5	TSS200	rs554848608; rs200849372; rs576316967
	cg25340688	VTRNA2-1	vault RNA 2-1	-0.38939399	0.000431041	5	TSS200	
	cg07753967	FRG1B	FSHD region gene 1 family member B	-0.29663176	0.000075	20	TSS1500	rs541560430; rs561838876; rs571919670; rs4635595; rs560582895
	cg18797653	VTRNA2-1	Vault RNA 2-1	-0.38086156	0.000301025	5	TSS1500	
	cg26896946	VTRNA2-1	Vault RNA 2-1	-0.35137256	0.000471807	5	TSS200	rs559858088; rs527487348
	cg06536614	VTRNA2-1	Vault RNA 2-1	-0.35887157	0.000473916	5	TSS200	rs577302824; rs533771522
	cg15971518	PRG2	Proteoglycan 2, pro eosinophil major basic protein	-0.33594632	0.00147907	11	TSS1500	rs549384200
	cg14815891	FRG1B	FSHD region gene 1 family member B	-0.23340448	0.000818547	20	Body	rs548363498; rs568541696; rs533898528; rs547808033

*CHR, chromosome; SNP, single nucleotide polymorphism.*

### Ontological Analysis of Differentially Methylated Genes of Whole Blood From Patients With CR

In the context of cellular component (CC) pathways, the 7,098 differentially methylated sites could be connected to many genes with bioinformatics profiling via the GO and DAVID databases. These genes could be arranged in 10 groups: (1) cytoplasm (GO:0005737); (2) plasma membrane (GO:0005886); (3) postsynaptic density (GO:0014069); (4) dendritic spine (GO:0043197); (5) cell junction (GO:0030054); (6) sarcolemma (GO:0042383); (7) cytosol (GO:0005829); (8) synapse (GO:0045202); (9) endoplasmic reticulum (GO:0005783); and (10) cell cortex (GO:0005938). Statistical analyses showed that the extent of methylation had significant differences for all CC categories above between patients and controls, with the fold enrichment ranging from 1.2 to 3 (Supplementary Table 1).

In the context of biological processing systems (BP pathways), the differentially methylated sites were arranged in 10 groups, including (1) homophilic cell adhesion via plasma membrane adhesion molecules (GO:0007156); (2) signal transduction (GO:0007165); (3) sensory perception of sound (GO:0007605); (4) cell adhesion (GO:0007155); (5) positive regulation of GTPase activity (GO:0043547); (6) negative regulation of protein serine/threonine kinase activity (GO:0071901); (7) intracellular signal transduction (GO:0035556); (8) axon guidance (GO:0007411); (9) angiogenesis (GO:0001525); and (10) lamellipodium assembly (GO:0030032). The extent of methylation changes showed significant differences between the CR and the control groups for each BP category above. The fold enrichment among these sets of pathways ranged from 1.4 to 5.5 (Supplementary Table 2).

The differentially methylated genes were arranged into 10 groups in the context of molecular function (e.g., biochemical cascade): (1) protein binding (GO:0005515); (2) calcium ion binding (GO:0005509); (3) actin binding (GO:0003779); (4) protein homodimerization activity (GO:0042803); (5) identical protein binding (GO:0042802); (6) metal ion binding (GO:0046872); (7) 3′,5′-cyclic-nucleotide phosphodiesterase activity (GO:0004114); (8) ubiquitin protein ligase binding (GO:0031625); (9) GTPase activator activity (GO:0005096); and (10) protein tyrosine kinase activity (GO:0004713). The extent of differential methylation indicated significant differences for each category above. The fold enrichment ranged from over 1.1 to nearly 4.3 (Supplementary Table 3).

### Genomic Network Analysis of Differentially Methylated Genes in Whole Blood From the CR Group

With bioinformatics assessment via the KEGG database, the genes with differentially methylated sites in CR could be organized into five genomic networks in all in the context of potential biological interplays (Table 6). These included (1) circadian entrainment (hsa04713), (2) calcium signaling pathway (hsa04020), (3) insulin secretion (hsa04911), (4) dopaminergic synapse (hsa04728), and (5) vascular smooth muscle contraction (hsa04270). The extent of the differential methylation showed significant difference between the CR and control groups for each pathway above, with the fold enrichment ranging from over 1.5 to more than 2 (Table 6).

**TABLE 6:** Genomic networks with altered methylation in CR.

	**Pathway term**	**DMS involved**	**Total DMS**	***P*-value**	**FDR**	**Fold enriched**
1	hsa04713: Circadian entrainment	36	3.0848%	3.82E–06	0.0010	2.0682
2	hsa04020: Calcium signaling pathway	53	4.5416%	1.40E–04	0.0150	1.5813
3	hsa04911: Insulin secretion	30	2.5707%	1.82E–04	0.0150	1.8838
4	hsa04728: Dopaminergic synapse	40	3.4276%	2.35E–04	0.0150	1.6876
5	hsa04270: Vascular smooth muscle contraction	37	3.1705%	3.77E–04	0.0193	1.6933

### Validation of the Differentially Methylated CpG Loci

DNA from the peripheral blood samples of 30 subjects with CR and 30 non-CR controls were extracted to assess the accuracy of results tested with the pyrosequencing. The clinical characteristics of the two groups of subjects were analyzed (Table 7). We found that age (*P* = 0.039) and uric acid (*P* = 0.001) were significantly higher in the CR group than in the control group. Our study also indicated that albumin in the CR group was significantly lower than that in the control group (*P* = 0.045), which was consistent with previous literature. We utilized logistic regression analysis by forward LR in the validation cohort, and found that uric acid and albumin were different in CR and non-CR groups (Table 8). And in subgroup analysis, we discovered the difference in group of albumin 40 g/L and UA ≤ 350μmmol/L. However, the albumin more than 40 g/L and uric acid less than 350 μmmol/L were in normal range, and almostly of no clinical significance. Hence, we considered that these variables might not affect the results significantly. And then, the remaining clinical characteristics between the two groups were shown to have no significant difference (*P* > 0.05).

**TABLE 7:** Characteristics of patients in the validation sample.

**Index**	**Non-CR (*n* = 30)**	**CR (*n* = 30)**	**z/t/χ^2^**	***P*-value**
HsCRP (mg/dL)	1.820 (0.870, 6.898)	3.545 (1.058, 8.098)	–0.924	0.355
AST (μmol/L)	24.5 (18.0, 80.5)	28.0 (19.5, 142.0)	–0.266	0.790
HbA1c (%)	5.93 ± 0.77	6.31 ± 1.24	–1.437	0.157
Triglyceride (mg/dL)	1.571 ± 0.936	1.275 (0.885, 1.698)	0.159	0.875
Blood sugar (mmol/L)	5.459 ± 0.958	5.956 ± 1.846	–0.207	0.198
BUN (mmol/L)	5.544 ± 2.569	5.990 ± 2.067	–0.741	0.462
Age (years)	60.5 ± 10.7	66.3 ± 10.3	–2.110	0.039
Left ventricular ejection fraction (%)	61.6 ± 7.1	58.6 ± 10.0	1.342	0.185
ALT (μmol/L)	37.5 ± 34.6	36.6 ± 30.3	0.107	0.915
Uric acid (μmmol/L)	256.7 ± 147.6	349.1 ± 115.3	–2.701	0.001
PLT (10^9^/L)	195.7 ± 57.1	185.5 ± 65.8	0.643	0.523
Number of stents per patient	1.0 ± 0.8	1.5 ± 0.9	–2.064	0.043
TBIL (mmol/L)	14.71 ± 5.87	14.17 ± 10.06	–1.464	0.143
Albumin (g/L)	39.72 ± 3.88	37.69 ± 3.78	2.052	0.045
Cr (μmol/L)	74.71 ± 21.01	76.61 ± 19.29	–0.356	0.716
MPV (fl)	8.67 ± 1.18	8.21 ± 1.28	1.438	0.156
BMI (kg/m^2^)	24.235 ± 2.533	23.944 ± 2.606	0.438	0.663
Total cholesterol (mg/dL)	4.817 ± 1.289	4.398 ± 1.266	1.271	0.209
HDL (mg/dL)	0.982 ± 0.203	0.982 ± 0.315	0.000	> 0.1
LDL (mg/dL)	2.992 ± 1.118	2.685 ± 1.060	1.089	0.281
PCT (%)	0.172 ± 0.051	0.150 ± 0.044	1.820	0.074
Male gender, *n* (%)	8 (26.7)	7 (23.3)	0.089	0.766
Alcohol abuse, *n* (%)	24 (80)	22 (73.3)	0.373	0.542
Hypertension, *n* (%)	10 (33.3)	10 (33.3)	0.000	> 0.1
Diabetes, *n* (%)	26 (86.7)	21 (70.0)	2.455	0.117
Dyslipidemia, *n* (%)	16 (53.3)	18 (60.0)	0.271	0.602
Current smoking, *n* (%)	23 (76.7)	18 (60.0)	1.926	0.165

**TABLE 8:** Logistic regression analysis in the validation cohort.

	**B**	**SE**	**Wals**	***P*-value**	**Exp (B)**
Albumin	-0.205	0.092	5.028	0.025	0.814
Uric acid	0.008	0.003	6.751	0.009	1.008
Constant	5.499	3.402	2.612	0.106	244.485

**FIGURE 4:**
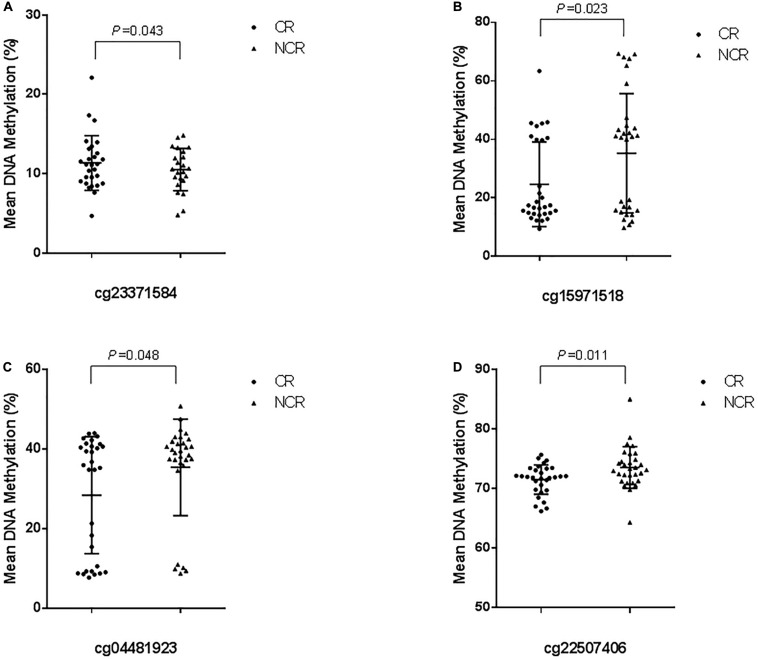
Cross-validation of DNA methylation with pyrosequencing. (The mean methylation: **(A)** cg23371584, CR vs. NCR: 10.598 ± 4.396% vs. 8.063 ± 5.085%, *P* = 0.043. **(B)** cg15971518, CR vs. NCR: 24.633 ± 14.445% vs. 35.263 ± 20.422%, *P* = 0.023. **(C)** cg04481923, CR vs. NCR: 28.401 ± 14.699% vs. 35.421 ± 12.142%, *P* = 0.048. **(D)** cg22507406, CR vs. NCR: 71.532 ± 2.474% vs. 73.576 ± 3.500%, *P* = 0.011).

The results of pyrosequencing of four sites (cg23371584, cg15971518, cg04481923, cg22507406) are as follows: for cg23371584, the methylation rates were 10.598 ± 4.396% in the CR group and 8.063 ± 5.085% in the control group (*P* = 0.043) (Figure 4A). The cg15971518 site had a methylation rate of 24.633 ± 14.445% in the CR group and 35.263 ± 20.422% in the control group, as shown by pyrosequencing (*P* = 0.023) (Figure 4B). Pyrosequencing revealed methylation of cg04481923 at 28.401 ± 14.699% in the CR group and 35.421 ± 12.142% in the control group (*P* = 0.048) (Figure 4C). The cg22507406 site had a methylation rate of 71.532 ± 2.474% in the CR group and 73.576 ± 3.500% in the control group (*P* = 0.011) (Figure 4D). The results above were correlated with the data from the Methylation850 BeadChip test.

**FIGURE 5:**
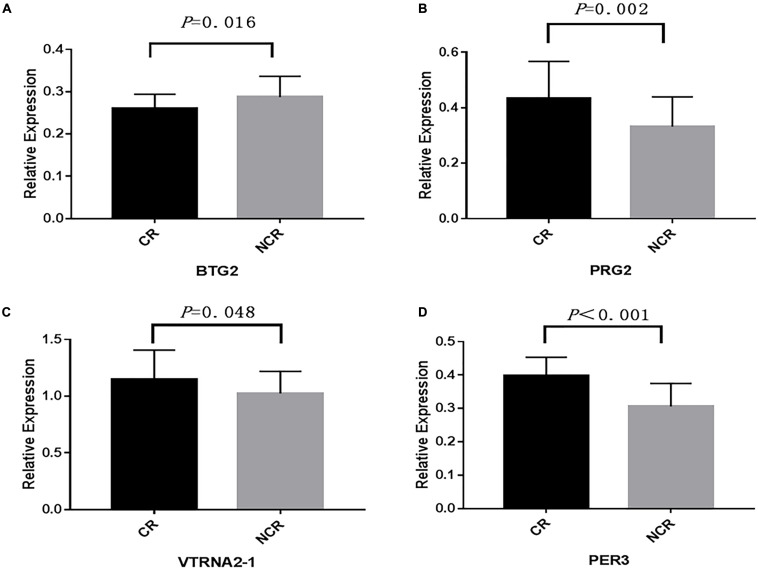
A comparison of the mRNA expression of four genes. (BTG2, PRG2, VTRNA2-1, PER3) between CR and non-CR. (The mean relative expression of mRNA: **(A)** BTG2, CR vs. NCR: 0.267 ± 0.031 vs. 0.293 ± 0.044, *P* = 0.016. **(B)** PRG2, CR vs. NCR: 0.423 ± 0.163 vs. 0.336 ± 0.096, *P* = 0.002. **(C)** VTRNA2-1, CR vs. NCR: 1.167 ± 0.285 vs. 1.007 ± 0.213, *P* = 0.048. **(D)** PER3, CR vs. NCR: 0.397 ± 0.056 vs. 0.308 ± 0.072, *P* < 0.001).

We assessed the relative mRNA expression of the genes (*BTG2*, *PRG2*, *VTRNA2-1*, *PER3*) corresponding to the four loci above through qRT-PCR. The results are as follows: *BTG2* mRNA expression was decreased when patients suffered from a poor clopidogrel response (*P* = 0.016) (Figure 5A). The mRNA expression of *PRG2* (*P* = 0.002) (Figure 5B), *VTRNA2-1* (*P* = 0.048) (Figure 5C), and *PER3* (*P* < 0.001) (Figure 5D) was increased in the CR group.

## Discussion

DNA methylation is a kind of epigenetic modification. Approximately 70% of CpG (cytosine-phosphate-guanine) dinucleotides are methylated in mammalian genomes. A methyl group is bound to the fifth carbon of the cytosine DNA base (5-mC) at the CpG site with a covalent link (Stratton et al., 2019). The CpG sites are relatively more common at the promoter regions of genes. Transcription factors frequently bind to 5-mC. Therefore, when methylation hinders transcription factors binding to 5-mC in CpG islands (CGIs), it may lead to gene silencing. DNA methylation will affect the expression of downstream DNA without altering the sequences, which is an important feature of epigenetics. Hypomethylation of CGIs may enhance the expression of genes, while hypermethylation is related to transcriptional silencing of gene expression (Takai and Jones, 2002). The mechanisms for gene repression include direct inhibition of transcription factor binding (methylated cytosines or methylcytosine binding proteins) and indirect inhibition involving methylcytosine-binding proteins and histone deacetylase complexes (Prasher et al., 2019). In particular, methylation levels might be significantly transformed in the context of disease conditions (Attwood et al., 2002). Differential DNA methylation of peripheral blood and tissues is reported in various diseases, including intestinal disease (Jorgensen and Ro, 2019), adrenocortical cancer (Legendre et al., 2016), breast cancer (Li et al., 2018), Parkinson’s disease (Chuang et al., 2017), and systemic lupus erythaematosus (Zhu et al., 2016). Moreover, DNA methylation plays great effect on the cardiovascular system. Recent epigenetic researches have revealed that DNA methylation is associated with susceptibility to hypertension (Kato et al., 2015; Demura and Saijoh, 2017), incident CAD or myocardial infarction (Agha et al., 2019), recovery after myocardial infarction (Rask-Andersen et al., 2016), and idiopathic dilated cardiomyopathy (Haas et al., 2013). DNA methylation has been rapidly elucidated in recent years, which will further contribute to our knowledge of CR.

Clopidogrel is a commonly used antiplatelet drug for patients with CAD, inducing antiplatelet aggregation by antagonizing the P2Y12 receptor. P2Y12 is a promising antiplatelet target, but due to constitutive activation of the P2Y12 receptor, CR commonly occurs. New P2Y12 inhibitors, such as ticagrelor and prasugrel, are recommended over clopidogrel in ACS patients undergoing PCI (Valgimigli et al., 2018). However, for East Asian patients with ACS, clopidogrel might be better than the new P2Y12 inhibitors because multiple studies have confirmed that treatment with ticagrelor or prasugrel has higher bleeding or efficacy endpoint event rates than treatment with clopidogrel in the East Asian population (Goto et al., 2015; Wiviott and Steg, 2015; Kang et al., 2018). After being absorbed through the intestine, clopidogrel is metabolized into the active ingredient through the liver, enters the circulation and binds to the P2Y12 receptor of the platelet. In this process, emerging data have indicated that DNA methylation plays a potential effect on the absorption and onset of the effects of clopidogrel. Related genes include PON1, ABCB1, and P2Y12. Our previous research found that the DNA methylation located in the CpG4 of PON1 promoter might increase the risk of CR in patients with dyslipidaemia (Su et al., 2019), while the methylation levels of the ABCB1 promoter had no relationship with CR (Su et al., 2017). Another study based on the Chinese population found that hypomethylation of the P2Y12 promoter was associated with higher platelet reactivity (Li et al., 2016). Moreover, a study from Spain demonstrated that hypomethylation of cg03548645 led to increased expression of TRAF3 (TNF receptor-associated factor 3), which was associated with CR (Gallego-Fabrega et al., 2016). In the context of these data, DNA methylation in genes might be one of the latent causes of CR. Whole-genome DNA methylation pattern studies may help us explore more potential sites, which may identify possible biomarkers and functional pathways.

The BeadChip assay system theoretically reports methylation changes across the whole genome. In this study, 7,098 differentially methylated sites were identified, with 979 sites being significantly hypermethylated and 6,199 sites hypomethylated in the subjects with CR relative to the non-CR controls. The ratio of whole hyper- to hypomethylated sites was 0.16. The ratio of the promoter region’s hypermethylated to hypomethylated sites was 0.43 (644/1,490), which confirms that decreased methylation occurs in slightly more sites than increased methylation. In our study, the data show that DNA methylation alterations in CR can be found in genes that are not associated with the pharmacokinetics and pharmacodynamics of clopidogrel. The bioinformatics profile demonstrated that the hyper- or hypomethylated sites can be linked to functional genes. Various genes (LILRA3, BTG2, PITX1, NDRG4, ARNT2, ESPNL, SPEG, SULT1A1, PTPN21, SDC2, FRG1B, VTRNA2-1, PRG2) have been related to CR among the top 10 hypomethylated genes and top 10 hypermethylated genes (Table 5). The discovery above supports the view that multiple epigenetic or genetic factors are participants in numerous cellular processes and are also related to some complex human conditions (Feinberg, 2010), including CR. Genetic testing has been used to distinguish the type of clopidogrel metabolism and assess the prognosis (Johnson et al., 2017; Moon et al., 2018), especially among Asians, where the proportion of patients carrying the CYP2C19^∗^2 allele is > 50% (Sun et al., 2016). The detection of differentially methylated genes has the potential to become a similar predictor. We used pyrosequencing and PCR amplification for validation, confirming that four differentially methylated CpG loci (cg23371584, cg15971518, cg04481923, cg22507406) were associated with the risk of CR. Overall, methylation data of the selected loci from the pyrosequencing method are consistent with the results from the BeadChip analysis. CpG cg23371584 maps to the BTG2 (B-cell translocation gene 2) gene, which is an antiproliferative gene encoding 158 amino acids. Hypermethylation of CpG cg23371584 may inhibit the expression of BTG2. CpG cg15971518 maps to the PRG2 (proteoglycan 2) gene, which is a pro-eosinophil major basic protein. CpG cg04481923 maps to the VTRNA2-1 (vault RNA 2-1) gene. CpG cg22507406 maps to the PER3 (period circadian regulator 3) gene, with a role in circadian rhythms. Platelet activity has circadian rhythm characteristics (Sibbing et al., 2016), which may be a clue to connect the PER3 gene with CR.

Based on the gene KEGG enrichment analysis results, five genomic networks are involved, as shown in Table 6: circadian entrainment, the calcium signaling pathway, insulin secretion, dopaminergic synapses, and vascular smooth muscle contraction. Circadian entrainment is associated with CR. Mouse studies have confirmed that circadian fluctuations in platelet functions are regulated by circadian clock proteins (Ohkura et al., 2009), and CLOCK mutations might be involved in platelet hyper aggregability during the whole day. Similarly, hypermethylation (CpG cg22507406) reduces the expression of PER3, which may be a circadian mechanism of CR. Moreover, insulin secretion reveals a new mechanism of CR. Among related genes, the GNAS (gene encodes the heterotrimeric Gs protein alpha-subunit) gene is a vital regulator in insulin secretion of pancreatic β-cells (Taneera et al., 2019). Epigenetic regulation (Tasnim et al., 2014; Sucharita et al., 2018) has been suggested to be profoundly involved in the pathogenicity of insulin resistance and T2D. CR may be closely related to pancreatic β-cell function and T2D, and methylation of the genes identified is a potential topic to explore the mechanisms of CR and insulin resistance. Notably, another study showed that DNA methylation levels exhibited relatively weak associations with neighboring gene expression levels across individuals (Van Eijk et al., 2012), which may be related to insufficient sample size. Therefore, we must be cautious about interpreting the results above, and a larger sample size study is needed to further clarify the correlation between the above pathways/genes and CR.

In addition, epigenetic modifications (histone marks, DNA methylation, and noncoding RNAs) have recently been a research hotspot in biomedicine. Evidence has indicated that epigenetic changes are responsible for many diseases and can provide new ideas for the treatment of diseases. For instance, histone deacetylase inhibition has been proposed to treat human heart failure (Jeong et al., 2018). Epigenetic mechanisms in CR have been explored in recent years, especially in the field of noncoding RNA and DNA methylation. There are almost no data on histone modification associated with CR. In terms of noncoding RNAs, lncRNA metallothionein 1 pseudogene 3 (MT1P3) and miR-126 might be the reason for CR in T2D patients (Zhou et al., 2019). Moreover, the differential expression of miR-15b-5p and miR-26a-5p in platelets is in connection with variability in clopidogrel response and reactivity of platelet (Freitas et al., 2016). In terms of methylation, the methylation of individual genes with specific functions, such as PON1, ABCB1, P2Y12, and TRAF3, has been extensively studied. Based on previous research, our study further elucidated the epigenetic features of CR from the analysis of methylation of peripheral blood genes in patients with a low clopidogrel response, revealing more genes and pathways related to CR. In epigenetic research, N6-methyladenosine (m6A) has recently been the new focus of mechanistic research and is considered to be pivotal in RNA cleavage, and mRNA translation, gene expression. Moreover, m6A is the most abundant mRNA post-transcriptional modification (Wang et al., 2015). We speculate that m6A may have an effect on CR, which needs further exploration.

This study is the first to demonstrate global hypermethylation and hypomethylation of whole blood genes in CR, revealing relevant methylation sites and possible intrinsic gene interaction pathways, which has certain guiding significance for the mechanistic study of CR. However, there were still some limitations that need to be addressed. First, the study population had certain geographical restrictions, and the sample size needs to be expanded. Second, we validated only four CpG loci (cg23371584, cg15971518, cg04481923, cg22507406) by the pyrosequencing method. Finally, advanced mechanistic validation based on cellular or animal experiments needs to be further implemented to explore internal mechanisms. However, this mechanistic study of CR clarified the methylation characteristics of genes in whole blood, which is important.

## Conclusion

In conclusion, our study provides the first set of human data suggesting differential DNA methylation at various site of the whole blood genome in subjects with CR compared to the non-CR controls and demonstrates that the different methylation changes of cg23371584, cg15971518, cg04481923, and cg22507406 are associated with CR. The relative mRNA expression of the four genes corresponding to the loci above was also associated with CR, suggesting that the change in DNA methylation may lead to different expression of the four genes, eventually resulting in CR. Furthermore, bioinformatics analysis showed that multiple of the differentially methylated locis are related to molecular pathways, genes, promoters and that are vital for metabolic and biological regulation of circadian entrainment, insulin secretion, and so on. In the future, expanding the sample size in multicentre studies with more comprehensive plans and empirical approaches will help us further explore the possible epigenetic mechanisms of CR.

## Data Availability Statement

The raw data supporting the conclusions of this article will be made available by the authors, without undue reservation, to any qualified researcher.

## Ethics Statement

The studies involving human participants were reviewed and approved by the Ethics Committee of Ningbo First Hospital. The patients/participants provided their written informed consent to participate in this study. Written informed consent was obtained from the individual(s) for the publication of any potentially identifiable images or data included in this article.

## Author Contributions

JS, JY, HX, LJ, and XC designed the experiments. JL, ZX, QY, JY, NZ, YL, JZ, and JS carried out the experiments. JS, QY, and LJ analyzed the experimental results. JY and QY wrote the manuscript. JS and XC helped project administration and manuscript review and editing. All authors contributed to the article and approved the submitted version.

## Conflict of Interest

The authors declare that the research was conducted in the absence of any commercial or financial relationships that could be construed as a potential conflict of interest.
