# Supplementary material for: Clopidogrel Resistance Is Associated With DNA Methylation of Genes From Whole Blood of Humans
Source: Front Genet. 2021 Jan 15;11:583215. doi: 10.3389/fgene.2020.583215 (PMC7844369; doi:10.3389/fgene.2020.583215)
Supplement: Supplementary file 1 [file Data_Sheet_1.docx]

Supplemental Table 1 Relevance of differentially methylated sites to cellular component (CC) genes.

| Pathway list | Pathway Term | DMS involved | total DMS | Genes | P Value | Fold Enrichment | FDR |
| --- | --- | --- | --- | --- | --- | --- | --- |
| #1 | GO:0005737~cytoplasm | 572 | 31.6372 | FLYWCH1, RBPMS2, FHIT, XRCC3, PTGS2, RPL14, SCN3A, RANGAP1, ZNF638, AMOTL1, CIAPIN1, CCDC141, RAE1, PPP1R1B, SMOX, TBPL1, MAP2K5, SH3GL3, MDH1B, PTPRM, MAGI2, TTC7A, MAGI1, DCDC2, SCYL3, MECOM, BTBD9, RPTOR, ZNF500, CTNNA2, GRB10, NME2, PPP1CA, HNF4A, SIPA1L1, MC2R, RYR1, ROR1, CMIP, GRB14, RALGPS2, RALGPS1, PABPC4, DUSP10, PRRC2A, BCL2L1, DAAM1, ASL, CTIF, KBTBD4, ZAP70, C19ORF24, TCF3, DNMT3B, UNC45B, SGIP1, RPS24, SPOP, DVL2, ZC3H14, ODC1, DNMT3A, DUSP22, PCDH15, UBE2L3, GAS7, ATM, SYNE1, SYNE2, PARP9, AKNAD1, TNK2, BTBD11, GAS2L3, KCNAB2, VIPAS39, KCNAB1, FGF9, TCOF1, TP63, LRRC15, MSRA, MGRN1, HSF1, NUBP2, MAPT, DPP8, USH2A, ZP3, MICAL3, TP53, FLNC, ELMO2, FMN1, EPB41L3, WDR47, NCK2, EPB41L1, CLIC5, SRGAP3, USH1C, KIAA1217, GUCY1B3, GNAS, USP25, EEF1D, FGGY, CREM, NBPF20, EPB41L4A, ZNF365, ZNF655, TRIM10, NECAB3, AMPH, SRRT, MTMR3, EZR, SORBS1, LRRTM4, SNRK, DGKD, PTK2B, EXOC4, DHX16, TBC1D4, TRIP12, PDK2, UPF2, SHMT2, BRD2, NUB1, NBPF10, AIMP1, DGKH, WWTR1, TP73, CDH13, PKNOX1, RPS6KA2, RASSF1, CHTF18, SPG11, KATNAL2, HPGD, CMTM3, ENAH, STK31, GMPR2, ZAK, EIF5, KNTC1, LMO7, CNOT1, IL15 | 1.55E-09 | 1.2277 | 2.36E-06 |
| #2 | GO:0005886~plasma membrane | 461 | 25.4978 | SGMS2, SCN3A, SLC9A3, C6ORF25, ITSN1, AGTR1, KCNK9, GNG4, CDH24, CDH23, MAGI2, PTPRF, TTC7A, CLCA4, MAGI1, GRIN2A, CDHR3, PTPRO, SSTR5, GRB10, PPP1CA, BACE1, MC2R, RYR1, ROR1, SLC30A10, GRB14, KLRC1, RALGPS2, RALGPS1, CACNB2, ATP12A, DAAM1, SLC29A4, ZAP70, SLC28A1, SLC28A3, SGIP1, DVL2, PPFIBP1, ATP11A, CD300C, PCDH15, DAGLB, KCNJ5, ARF1, RAPSN, PKP3, RGS6, CHRND, TNK2, SYTL1, GNAZ, LDLR, KCNAB2, KCNAB1, DPP10, CRCP, KCNK10, MGRN1, MAPT, ANGPT1, SLC22A6, DPP6, DPEP1, ZP3, CMKLR1, ARHGEF7, SLC22A7, MICAL3, SDK1, IL6R, FLNC, FMN1, EPB41L3, FMN2, EPB41L1, GNB1, DOK7, KCNH6, USH1C, GNAS, GUCY1B3, PARVB, ENOX1, ALPL, GPR63, C2ORF88, IGSF11, EZR, ACSL1, SORBS1, HPSE2, DGKD, PCDHB16, EXOC4, EXOC2, AXL, DGKH, TMPRSS6, CDH13, RASSF1, CDH19, CTNS, SPG11, LRP5, SLC8A3, ENAH, KCNC4, SLC13A5, SLC44A2, SLCO1B7, GRIK2, SLC44A5, TSPAN4, GRIK4, EIF5, KNTC1, TSPAN9, KANK1, SLC7A7, NRCAM, IFT122, ANK1, SLC2A5, UNC5B, ANK3, FRS2, INSR, FLVCR2, CIB1, EGFR, KCND3, BAIAP2, SLC34A1, IL11RA, SLC34A2, HLA-F, NCAM1, ARRB2, ARRB1, RELN, MRAP, DRD2, ADORA2A, MRAS, NKAIN2, LRIG2, NKAIN3, ABCA3, OR2L13, PDE6B, P2RY6, PTK2, ECE1, TTYH2, OR51B5, TRAF6, PHACTR2, VSTM4, VAV3, SLC6A12, SLC6A13, CACNA1I, SPPL2B, VAV2, SLCO2B1, PDE6G, SHANK2, CACNA2D2, FURIN, EPS15, P2RX4 | 1.16E-08 | 1.2538 | 1.77E-05 |
| #3 | GO:0014069~postsynaptic density | 41 | 2.2677 | ARFGAP1, PALM, KCNAB2, GRIK2, DRD2, ADORA2A, CAMK2G, CLSTN1, CABP1, CPEB1, ADORA1, DTNBP1, NETO1, PLCB4, PTK2B, MAPT, SYN3, PDE4B, AXIN1, DLG1, ANKS1B, DLGAP1, BCR, MAGI2, BAIAP2, GRIN2A, SHANK2, PCLO, ITPR1, CTNNA2, P2RX4, EPB41L3, RGS20, ARF1, ARRB2, ARRB1, SIPA1L1, SPTBN1, NRGN, CACNA1C, KALRN | 9.07E-08 | 2.4974 | 1.38E-04 |
| #4 | GO:0043197~dendritic spine | 27 | 1.4934 | SLC8A3, PALM, DRD2, ASAP1, IGF2BP1, GIPC1, LPAR1, ADORA1, DTNBP1, CTTN, NR1D1, GPM6A, PDE4B, GRID2, ANKS1B, BAIAP2, STRN4, ASIC2, PTPRO, SHANK2, FARP1, P2RX4, PPP1CA, ARRB2, ARRB1, SIPA1L1, SEZ6 | 4.37E-07 | 3.0261 | 6.64E-04 |
| #5 | GO:0030054~cell junction | 70 | 3.8717 | SLC8A3, ENAH, KCNAB2, GABRB3, GRIK2, GRIK4, CLSTN1, GABBR1, FER, CPEB1, AMOTL1, ITSN1, ATP2B2, PRRT2, GRID2, ANO7, DSCAM, DLG1, CHRNA2, ANKS1B, STX1A, CTBP2, BCR, MAGI1, PTPRN2, GRIN2A, SDK1, NRXN1, PCLO, FARP1, COLQ, XIRP2, SIPA1L1, DOK7, SCIN, DSP, ERC2, MPST, PRKCZ, PARD3, CABP1, BCL2L1, DTNBP1, FAAP20, RIMS4, AMPH, NETO1, RGS12, SHISA9, LRRTM4, SYN3, LRRTM2, TES, DTNA, PHACTR1, ICA1, DLGAP1, GRIA4, SHANK2, TP73, RUFY3, P2RX4, CADPS2, GRIA2, RAPSN, RAP1A, CHRND, TJP2, NFIA, PUF60 | 1.14E-05 | 1.7093 | 0.0173 |
| #6 | GO:0042383~sarcolemma | 22 | 1.2168 | SLC8A3, KCND3, NOS1AP, TRIM72, FLNC, DTNBP1, GHRHR, VCAM1, SLC2A5, DYSF, ANK1, ANK3, RYR3, COL6A3, COL6A2, RYR1, SGCD, SCN5A, FKRP, CIB1, DTNA, DLG1 | 1.30E-05 | 2.9009 | 0.0197 |
| #7 | GO:0005829~cytosol | 361 | 19.9668 | FHIT, RPL17, SEC31A, RPL14, VPS54, RUSC2, PPP2R5C, PPCS, AURKA, RANGAP1, AMOTL1, ITSN1, PRKG1, SERPINE2, PPP1R1B, BTBD3, DYNC2H1, LSM5, SULT1A2, ERAP1, VPS13A, SMOX, CAB39, RAPGEF1, WWOX, MAP2K5, NWD1, DAB2IP, BCR, HKDC1, WNK1, WNK2, MECOM, FARP1, RPTOR, TANK, CTNNA2, FARP2, GRB10, NME2, PPP1CA, TRAPPC9, RGCC, GRB14, MPST, CHKA, NFKBIB, ASAP1, IGF2BP1, IGF2BP2, MYO9B, BCL2L1, ASL, DAAM1, EPHB2, NPHP4, ZAP70, AANAT, FBXO5, TCTN1, RAP1GAP2, PPP2R2C, UNC45B, BLNK, RPS24, DVL2, GNAT1, OSBPL5, ODC1, KIF3A, S100A16, ABR, ADAL, CHURC1-FNTB, UPB1, LGALS8, SMAD3, TKT, GAS2, CDC27, S100A13, TAX1BP1, DENND1B, RERG, DHFR, ARF1, PARP9, UBA3, RGS6, GNAZ, NRP1, KCNAB2, KCNAB1, BCAR1, MYO7A, AP1AR, PPARG, TP63, CRCP, LATS1, MCF2L, CAMKK1, BAG5, TRAPPC6A, MSRA, MGRN1, HSF1, MAPT, ZFAT, FGF1, ARHGEF4, DYNC1I1, STX1A, ARHGEF7, TP53, PDE10A, FLNC, ELMO2, FMN2, ATP6V1C2, NCK2, EPB41L1, GNB1, ADK, SRGAP3, USH1C, PLA2G6, GNAS, NUP107, EEF1D, STEAP2, PARVB, MAP3K12, PPFIA2, MYO5A, PARD3, POLR2F, RAP1GAP, TH, HK2, ZBTB16, SFN, MTMR3, SET, EZR, SORBS1, PDE1C, PTK2B, KLC1, PLIN3, PDE1A, EXOC4, IPCEF1, SULT1C2, EXOC2, BUB3, TRIP12, CEP131, UPF2, POLR3H, AIMP1, VTA1, WWTR1, SOD1, TP73, GBA3, AP2A2, PKNOX1, RPS6KA2, CHN1, CHN2, SPG11, HPGD, TJP2, ENAH | 1.36E-05 | 1.2205 | 0.0207 |
| #8 | GO:0045202~synapse | 35 | 1.9358 | ARFGAP1, PPFIA2, ENAH, GABRB3, MYO7A, MME, CPEB1, ITSN1, NRCAM, ATP2B2, SHISA9, RGS12, PRRT2, ARPC2, GRID2, DTNB, DTNA, DSCAM, EGFR, PHACTR1, DLGAP1, MAGI2, SDK1, PCDH15, ASIC1, PCLO, NCK2, RAPSN, ADGRV1, DOK7, CYFIP1, USH1C, APBB2, SPG11, MPST | 2.37E-05 | 2.1673 | 0.0360 |
| #9 | GO:0005783~endoplasmic reticulum | 109 | 6.0288 | SEC31A, PTGS2, FAM20A, B3GALT5, C6ORF25, SYNCRIP, UBQLN1, ADORA1, ATP2B2, ANK3, PLOD2, INSIG1, B3GALNT2, ERAP1, ELOVL7, SAR1A, CIB1, DDRGK1, NBAS, GRIN2A, MOGS, VASH1, HLA-F, PNPLA7, ADAMTS9, TRAPPC9, BACE2, PLA2G2A, CALR3, FAM172A, TPST2, PACS2, FGFR4, EXTL3, EEF1B2, CRELD2, FGFR3, MRAP, UBAC2, NRROS, UFSP2, VAC14, ATP11A, REEP1, FURIN, ATP2A2, KIAA0922, KCTD17, GHDC, AHCYL2, PDCD6, GNAZ, PCDHA2, ALG3, SULT2B1, PCDHA1, TRAPPC6A, SERAC1, EMID1, CALCRL, ANO7, KCNQ1, DLG1, MINPP1, KDELR2, AIFM3, ZDHHC6, ZP3, TP53, NRXN1, NLRP3, MCTP1, KIF1C, TRDN, NCK2, CEMIP, USP25, PRNP, EEF1D, SEZ6, SEC23B, MYO5A, SLC39A14, BRSK2, ZDHHC19, SEC62, CPED1, STAU2, VCAM1, RGMA, SET, PEX16, PYCARD, SLC39A7, ACSL3, SCN5A, NOX5, CREB3, AIMP1, TMBIM6, MAN1C1, ITPR1, PARK7, PROM1, PSMG1, SULF2, CASP12, SUMF1, LRP5 | 3.35E-05 | 1.4754 | 0.0509 |
| #10 | GO:0005938~cell cortex | 26 | 1.4381 | PARD3, USP2, MYO7A, CABP1, AKAP12, MYO9B, GIPC1, FER, ASTN2, PTK2, CTTN, PTK2B, FGF1, TRAF6, AXIN1, ARHGEF7, TLE6, ERMN, RAI14, FMN2, RGS20, FNBP1L, CLIC5, SCIN, RYR1, DST | 7.33E-05 | 2.3691 | 0.1114 |

Supplemental Table 2 Relevance of differentially methylated sites to biological process (BP) genes.

| Pathway list | Term | DMS involved | total DMS | Genes | P Value | Fold Enrichment | FDR |
| --- | --- | --- | --- | --- | --- | --- | --- |
| #1 | GO:0007156~homophilic cell adhesion via plasma membrane adhesion molecules | 37 | 2.0465 | CADM3, PCDHA2, PCDHA3, CLSTN1, PCDHGA9, PCDHGA8, PCDHGA7, PCDHGC4, PCDHGA6, PCDHGC3, PCDHA1, CDH4, PCDHGA1, PCDHGB1, PCDH1, ROBO1, PCDHB16, PCDHA11, CDH24, CDH23, DSCAM, KIRREL3, PCDHGA12, PCDHGA11, PTPRM, PCDHB3, PLXNB2, SDK1, PCDHGB7, CDHR3, PCDH9, PCDH15, PCDHGB5, NCR3, PCDHGB4, CDH13, CDH19 | 1.84E-07 | 2.5735 | 0.0003 |
| #2 | GO:0007165~signal transduction | 154 | 8.5177 | SLC22A17, SLC44A2, GABRB3, HBS1L, PPP2R5C, RANGAP1, IL15, RADIL, FGF12, PRKG1, VIPR2, ADORA1, ANK1, UNC5B, LILRA3, PPP1R1B, ANK3, PDE4A, ATF6B, PDE4B, PITPNC1, RAPGEF1, GNL1, CHRNA2, MAP2K5, EGFR, SH3GL3, PTPRK, CASKIN2, PTPRM, MAGI2, BCR, STK25, PLXNB1, PDE4C, IL21, TANK, STK3, LILRB2, CD38, GRB10, ARRB2, ARRB1, LRP12, PDE5A, STMN1, GRB14, KALRN, TRAF1, CHKA, NFKBIB, ARFRP1, MYO9B, SP110, IRAK4, RASAL1, PDE6B, NPHP4, DOCK1, NPM1, OR51B5, TRAF3, AXIN1, GNAT1, VAC14, ABR, CACNA1I, CD300C, VAV2, ATM, P2RX4, HIVEP3, RAP1A, CHRND, CLCN6, PLCXD2, PTGES3, NRP1, FGF9, PPARG, ARHGAP17, RASSF8, KCNK10, EDNRA, ARHGAP22, WISP1, PRMT2, WISP3, PAK4, ZFYVE16, RANBP1, PLCB1, FGF1, NRG2, AGAP3, AKT3, LTBR, NRXN2, ARHGEF7, NRXN3, ESR1, TLE3, PDE10A, INHA, NRXN1, NLRP3, HMGA2, ARHGAP26, NCK2, ACVR2B, PSD, GNB1, GRN, SRGAP3, INPP4B, MAPRE2, MCC, INPP4A, NRGN, EEF1D, CHL1, PRKCZ, RAP1GAP, CREM, AKAP9, NR3C1, SFN, LINGO1, SMOC2, IGF1R, RGS12, PTK2B, DGKD, PDE1C, BCL11B, PDE1A, PYCARD, PLA2R1, DTNA, AIMP1, SNX27, NF1, AXL, DPYSL5, RAF1, ASIC1, DRG2, ITPR1, GRIA2, RPS6KA2, TOM1L2, CHN1, CHN2, BCAR3 | 1.16E-06 | 1.4577 | 0.0022 |
| #3 | GO:0007605~sensory perception of sound | 31 | 1.7146 | GABRB3, THRB, TSPEAR, DIAPH1, STRC, MYO7A, TH, LRIG2, MBP, ATP2B2, FAM107B, COL11A2, KCNQ1, USH2A, CDH23, CNTN5, ASIC2, DCDC2, PCDH15, SOD1, TIMM8B, EML2, LOXHD1, SLC26A5, NAV2, CLIC5, ADGRV1, CEMIP, USH1C, ATP6V0A4, CACNA1D | 2.36E-06 | 2.5615 | 0.0044 |
| #4 | GO:0007155~cell adhesion | 73 | 4.0376 | NRP2, SPG7, OPCML, PCDHA2, PCDHA3, IGFBP7, BCAR1, CLSTN1, VTN, POSTN, EDIL3, PCDHA1, FER, MOG, DGCR2, WISP1, WISP3, ROBO1, COL12A1, DSCAM, CIB1, PTPRK, PTPRF, CNTN5, MAGI1, PCDHB3, SIGLEC11, AJAP1, CTNNA2, PCDHGB4, FARP2, NCAM1, PRKD2, NME2, LSAMP, CNTN1, VCAN, RELN, CNTN4, SEMA4D, ADAM12, DST, PARVB, CHL1, ROM1, PCDHGC4, PCDHGC3, CDH4, ITGBL1, VCAM1, IGSF11, SORBS1, SORBS2, COL6A3, ACAN, COL6A2, PCDHA11, THBS3, COL18A1, HAPLN1, TNXB, AIMP1, LPP, PODXL, PPFIBP1, ITGA3, TINAG, COL5A1, CDH13, ATP2A2, THEMIS2, NTM, HABP2 | 3.05E-06 | 1.7478 | 0.0058 |
| #5 | GO:0043547~positive regulation of GTPase activity | 83 | 4.5907 | FGF5, FGF9, RGL3, RANGAP1, ARHGAP17, ITSN1, TBC1D19, MCF2L, ARHGAP22, RANBP1, ANGPT1, AGAP1, DOCK10, FGF1, PLCB1, RAPGEF1, NRG2, FRS2, AGAP3, EGFR, ARHGEF4, DAB2IP, BCR, ARHGEF7, PLXNB1, GRIN2A, ECT2, FARP1, ARHGAP26, FARP2, NCAM1, NCK2, PLCE1, PSD, FNBP1L, ARRB1, SRGAP3, GNAS, SEMA4D, SPATA13, KALRN, SPTB, ARFGAP1, CAV2, SNX18, RALGPS2, FGFR4, FGFR3, RALGPS1, RAP1GAP, CAMK2G, ASAP1, AKAP9, MYO9B, CYTH2, NPRL3, LLGL2, ADCYAP1, AMPH, RASAL1, PTK2, DOCK1, RGS12, RAP1GAP2, ELMOD1, AXIN1, DVL2, OBSCN, GNAO1, ABR, VAV3, NF1, VAV2, DENND1B, DIS3, RGS20, TSC2, RGS6, CHN1, RAP1A, SPTBN1, CHN2, BCAR3 | 1.37E-05 | 1.6144 | 0.0258 |
| #6 | GO:0071901~negative regulation of protein serine/threonine kinase activity | 9 | 0.4978 | DAB2IP, PPP1R1B, PRKAG2, PYCARD, WNK1, SFN, DTNBP1, LRP5, CIB1 | 8.74E-05 | 5.4948 | 0.1647 |
| #7 | GO:0035556~intracellular signal transduction | 61 | 3.3739 | ADCY1, NRG3, ZAK, PLEKHM1, ADCY5, PRKAG2, FER, ITSN1, MCF2L, PLCB4, PPP1R1B, CAB39, PLCB1, NRG2, AKT3, ARHGEF4, BCR, PLXNB1, ZP3, ARHGEF7, WNK1, DCDC2, WNK2, DAPK2, DAPK3, ECT2, STK3, FMN2, PRKD2, LAT2, TNS1, GUCY1B3, STMN1, MAP3K12, KALRN, PRKCZ, RALGPS1, DRD2, MYO9B, RASAL1, CORO2A, STK32B, STK32A, SNRK, DGKD, ASB10, ZAP70, BLNK, DVL2, ABR, RAF1, DGKH, TMPRSS6, CCDC68, RPS6KA2, RASSF1, PRKAR1A, CHN1, RGS6, CHN2, APBB2 | 9.30E-05 | 1.6634 | 0.1753 |
| #8 | GO:0007411~axon guidance | 30 | 1.6593 | NRP2, ENAH, NRP1, CDH4, EPHB2, PTK2, EZR, ANK3, ROBO1, MEG3, NKX2-1, KLF7, ZNF280D, NRXN3, DPYSL5, NFASC, NRXN1, PTPRO, SLIT2, SLIT3, NCAM1, ISPD, SPTBN1, MAPK8IP3, CNTN4, RELN, APBB2, BMP7, CHL1, SPTB | 2.18E-04 | 2.0735 | 0.4099 |
| #9 | GO:0001525~angiogenesis | 38 | 2.1018 | NRP2, NRP1, PTGS2, FGF9, MMP2, EPHB2, NRCAM, WARS, ARHGAP22, PTK2, HOXA3, UNC5B, PTK2B, TGFA, ERAP1, ANGPT1, CALCRL, FGF1, RAMP1, CIB1, PTPRB, COL18A1, DAB2IP, NOX5, VAV3, AIMP1, NRXN3, HSPG2, NRXN1, VAV2, TMPRSS6, VASH1, PRKD2, PKNOX1, ID1, PDCD6, SEMA4A, ADAM15 | 2.42E-04 | 1.8727 | 0.4551 |
| #10 | GO:0030032~lamellipodium assembly | 11 | 0.6084 | ABLIM1, ARHGEF4, CDH13, NCK2, VAV3, ARHGEF7, CYFIP1, VAV2, PTPRO, SPATA13, PARVB | 2.80E-04 | 3.8995 | 0.5264 |

Supplemental Table 3 Relevance of differentially methylated sites to molecular function (MF) genes.

| Pathway list | Pathway Term | DMS involved | total DMS | Genes | P Value | Fold Enrichment | FDR |
| --- | --- | --- | --- | --- | --- | --- | --- |
| #1 | GO:0005515~protein binding | 927 | 51.2721 | RBPMS2, VPS54, PPP2R5C, SYNCRIP, RORC, MED23, RADIL, ITSN1, AMOTL1, AGTR1, SERPINE2, BTBD1, ZNF772, CAB39, TBPL1, CDH23, SH3GL3, PCID2, GRIN2A, SERPING1, SCYL3, MECOM, PPP1CA, MC2R, BACE1, ROR1, RYR1, CMIP, KLRC1, NFKBIB, PABPC4, CACNB2, BCL2L1, ASL, NCAPG2, ZAP70, GCSH, PPP2R2C, SGIP1, SPOP, ZC3H14, SSSCA1, KIF3A, ZC3H18, MOBP, ESRRG, ATP11A, TMEM110, UPK3A, UBE2L3, GAS7, ATM, PARP9, ARF1, RGS6, PHYKPL, TAF1C, BCKDK, LDLR, VIPAS39, GAR1, TCOF1, TP63, C19ORF47, MGRN1, SLC22A6, FGF1, DPEP1, BRD9, HYAL3, SLC22A7, TP53, MRPL9, LRRC29, IL6R, ELMO2, VPS8, EPB41L3, EPB41L1, ZNF747, CLIC5, USP20, ZNF550, VCAN, NUP107, NCAPH2, EEF1D, USP25, PARVB, C2ORF88, ZNF365, TRIM10, NECAB3, PCOLCE, AMPH, ZNF169, MTMR3, SRRT, SORBS1, SORBS2, EXOC4, DHX16, ZNF750, BRMS1L, EXOC2, TXNL4B, BRD2, UPF2, LMX1B, AIMP1, AXL, NTNG1, SF3A1, TP73, USP49, MEGF6, LRP5, JDP2, ENAH, LMO4, ZNF580, KNTC1, IL15, IFT122, ATG5, MDFIC, PACSIN2, TIA1, ATG7, ATF6B, FRS2, CIB1, POU2AF1, BAIAP2, CHID1, ARRB2, ARRB1, MIB2, STMN1, PDCD6IP, SPATA13, SPTB, ARFGAP1, ADAMTSL2, ADORA2A, LPAL2, PTK2, FBXW7, P2RY6, SMARCB1, ARPC2, TTYH2, HELLS, PHACTR2, VAC14, CREBZF, SPPL2B, EHMT2, FURIN, EPS15, P2RX4, DYRK1A, EBF1, ST8SIA5, SYT17, PC, ATP7B, PTGES3, MAD1L1, CLSTN1, GABBR1, PAX2, SKAP1, THADA, SPRY4, MBP, WARS, ACOT7, PRMT2, PAX8, EDC3, E4F1, VPS16, LRRFIP1, INO80E, EPN3, PPP2R1A, ATPAF2, DDB1, C17ORF62, MCU, STXBP4, SARNP, KIF1C, TRDN, KIF1B, UQCRH, SLC41A3, MAPRE2, MCC, CA2, MAPRE3, IFT88, EPN1, PMEPA1, EPN2, PPP2R2A, SNX18, HAUS1, GRAMD3, FAM13C, RGMA, FCN2, GTF3C5, GTF3C1, SCN5A, SCNN1A, CNNM3, GMDS, SNX27, SCRN1, RAF1, BCS1L, CUL4A, ID1, PRR13, NELL1, AURKA, LPAR1, PRKG1, ATP2B2, WWP2, ERAP1, SULT1A2, VPS13A, WWOX, PLD1, PRAME, BCR, WNK1, RAD9A, TANK, PRDM7, LRCH1, COLQ, LCA5, RGCC, TRMT12, RAB15, PIAS2, MYH7B, KAZN, GPATCH2L, RALYL, NEK1, MME, MYO9B, EPHB2, FBXO5, LHX6, RAP1GAP2, LHX9, FCHO2, BLNK, PLAT, C7ORF50, ZBTB48, IKZF3, UFSP2, TPD52L1, TKT, NTRK3, RNF8, METTL13, C1ORF116, ATP2A2, MLX, ATP2A1, DLX4, HOPX, BEGAIN, NCOR1, TCF12, ZFHX3, NCOR2, PPARD, NRP1, MYO7A, PPARG, ZEB2, ZEB1, KCNIP1, TRAPPC6A, GOLGA7, CCNY, SLC24A1, TGFA, ATP6V0D1, STX1A, CTBP2, APTX, MBD2, LIN54, SEMA4D, CTDP1, MAP3K12, SEMA4A, PPFIA2, KIZ, PARD3, RAP1GAP, TH, ZBTB16, NR1D1, GPM6A, KLC1, BCL11B, PEX16, PLIN3, SULT1C2, BCL6, PRAP1, PEX10, TES, DLGAP1, HSPG2, SOD1, PROM1, ERP44, TEX14, AP2A2, GRIA2, UBTF, COG8, TOM1L2, RAB35, CHN1, CHN2 | 1.97229E-11 | 1.1544 | 3.24541E-08 |
| #2 | GO:0005509~calcium ion binding | 101 | 5.5863 | NELL1, EDIL3, ITSN1, TTN, ATP2B2, DYSF, CDH24, CDH23, CIB1, PCDHGA12, EFCAB11, PCDHGA11, NCALD, CRTAC1, CDHR3, ACTN3, PCLO, PITPNM2, RYR3, PLA2G2A, RYR1, CALR3, DST, CRELD2, ASTN2, CRB1, EFCAB2, PCDHA11, GPD2, TESC, S100A16, PCDH15, CPS1, S100A13, EPS15, ATP2A2, ATP2A1, RHOT1, SYT13, SYT17, PDCD6, SYTL1, CACNA1A, CACNA1B, HABP2, PCDHA2, LDLR, PCDHA3, CLSTN1, PCDHGA9, PCDHGA8, PCDHGA7, PCDHGA6, PCDHA1, KCNIP1, PCDHGA1, PCDHGB1, PCDH1, PLCB4, SLC24A4, SLC24A2, SLC24A1, TPO, PLCB1, PCDHB3, RPH3AL, PCDHGB7, PCDH9, MMP17, NRXN1, PCDHGB5, SLIT2, SLIT3, MCTP1, PCDHGB4, ADGRV1, SCIN, VCAN, MYO5A, ADAMTS13, CABP1, PCDHGC4, PCDHGC3, NECAB3, CDH4, SMOC2, PCDHB16, ACAN, THBS3, PNLIPRP2, NOX5, HSPG2, MAN1C1, ITPR1, CDH13, EYS, SULF2, CDH19, MCFD2, ANXA13, MEGF6 | 1.10973E-05 | 1.5411 | 0.0183 |
| #3 | GO:0003779~actin binding | 49 | 2.7102 | GAS2L3, LRRC10, ENAH, VPS16, MKL1, BCL7B, KCNMA1, NCALD, MICAL3, CORO7, ACTN3, FMN1, TNNT2, FMN2, TNNT3, EPB41L3, EPB41L1, TNS1, XIRP2, MIB2, SCIN, DST, PARVB, MYH7B, SPTB, MYO5A, ABLIM1, ABLIM2, DIAPH1, CALD1, MYO9B, GIPC1, DAAM1, DAAM2, PTK2, EZR, SORBS1, PACRG, AFAP1, PHACTR1, PHACTR2, PHACTR3, MOBP, SYNE1, SYNE2, NEB, MYPN, SPTBN1, MYLK | 1.20805E-05 | 1.9283 | 0.0199 |
| #4 | GO:0042803~protein homodimerization activity | 100 | 5.5310 | GNPTG, RBPMS2, CADM3, PTGS2, GRIK2, SLC16A1, KCNK9, ATG7, PDGFC, DAB2IP, STK25, SLC34A1, ACTN3, MECOM, PTPRO, TBC1D22A, HNF4A, PDCD6IP, DST, CAV2, CHKA, MCL1, DRD2, RRAGA, GIPC1, MYO9B, BCL2L1, SRF, ECE1, NPM1, HARS2, SCARB1, SLC30A8, TCF3, AXIN1, ODC1, IKZF3, TESC, CARD9, S100A16, SPPL2B, SMAD3, TKT, TPD52L1, WRN, LHPP, S100A13, RNF8, P2RX4, SYNE1, MLX, ATP2A1, TSC2, PDCD6, TCF12, KYNU, MYO7A, HLCS, ASGR1, ACOT7, PRMT2, MGLL, SLC22A6, LRRFIP1, PLCB1, ANO6, USH2A, TERT, CTBP2, IL6R, ECT2, DAPK3, SLIT2, NCR3, TARBP2, SLC26A5, MAP3K12, RAP1GAP, CAMK2G, CD247, CALCOCO2, ZNF365, ZBTB16, DGKD, MYOM3, PYCARD, CEP131, PDK2, MSH3, AIMP1, CREB3, NPR3, SOD1, WWTR1, PARK7, CDH13, SP1, ID1, SUMF1, HPGD | 3.87808E-05 | 1.4987 | 0.0638 |
| #5 | GO:0042802~identical protein binding | 99 | 5.4757 | FHIT, VTN, MBIP, MCM10, TTN, UBQLN1, AMOTL1, CLK3, PACSIN2, ROBO1, SH3GL3, EGFR, DDX39A, DAB2IP, PTPRM, BAIAP2, UHRF1, NMNAT1, MPST, TRAF1, RALYL, DRD2, ADORA2A, MIPOL1, CTPS1, BCL2L1, ASL, DAAM1, EPHB2, FBXW7, MNS1, TRAF6, AXIN1, COL18A1, DVL2, ZBTB48, DNMT3A, IKZF3, CREBZF, SMAD3, TPD52L1, HGF, EPS15, CORO1B, UBA3, DYRK1A, AIRE, KCTD17, TNK2, PHYKPL, PDCD6, PUF60, SCHIP1, NIF3L1, LDLR, PPARG, TP63, HSF1, EDC3, LTBR, PFKL, SDK1, TP53, SF1, ESR1, ADIPOR1, MCU, RB1, DAPK2, DAPK3, SLIT2, TARBP2, KLHL12, MAPRE2, PRNP, OAT, MAPRE3, CLDN18, USP2, USP4, CD247, SFN, ZBTB16, GRAMD3, PBLD, IGF1R, PYCARD, BCL6, SHMT2, GMDS, L3MBTL1, SUN2, RAF1, CBY1, SOD1, TP73, CPT1A, PARK7, ATXN3 | 0.0002 | 1.4461 | 0.2675 |
| #6 | GO:0046872~metal ion binding | 233 | 12.8872 | FLYWCH1, ADCY1, PTGS2, PLEKHM1, ADCY5, POSTN, CIAPIN1, ATP2B2, ZFP91, ZNF772, MAP2K5, CLCA4, ZNF48, MECOM, TANK, NME7, ZNF37A, ZNF500, NME2, PPP1CA, PITPNM2, PRDM9, ZNF382, MGAT5B, ZNF610, NEK1, ASAP1, MYO9B, ATP12A, GALNS, FBXO5, DNMT3B, ZC3H14, MGAT4B, GNAT1, COL18A1, PLAG1, DNMT3A, ZBTB48, KLF7, IKZF3, ZC3H18, ADAL, UPB1, SMYD3, ANKMY1, ZBTB40, TKT, AARSD1, DAGLB, TAX1BP1, ATP2A2, FAM170A, TRPS1, ATP2A1, TNK2, GNAZ, NRP2, NRP1, ZNF155, TP63, ZEB2, CPEB1, ZEB1, HPDL, ASGR1, GFI1B, ZNF682, NUBP2, ZFYVE9, ZNF680, ZFAT, AGAP1, COL11A2, AGAP3, DPEP1, KCNMA1, ZBTB20, ZNF283, AIFM3, TP53, RPH3AL, APTX, PDE10A, ZNF747, ADK, GNAS, GUCY1B3, ZNF550, ADAM12, STEAP2, ZNF484, ADAM15, ZMYND12, ALPL, USP2, USP4, ZBTB16, ZNF655, ERI3, FAAP20, CYB561D2, ZNF169, MTMR3, DGKD, SORBS2, PDE1C, BCL11B, PITRM1, PDE1A, ZNF286A, ZNF750, BCL6, PPP1R10, DGKH, SOD1, PCK2, TP73, ZSCAN30, RASSF1, SDHD, NARFL, CHN1, CHN2, ZNF462, SLC8A3, GMPR2, ZNF580, ZNF827, ZIC1, MCM10, CLYBL, PDE4A, PDE4B, PHOSPHO1, PPP4C, RNF31, KCND3, STK25, FIBCD1, PDE4C, TRERF1, TIMM8B, TATDN3, PLCE1, MIB2, PDE5A, RELN, KALRN, ARFGAP1, EXTL3, TSHZ1, TIMP2, RASAL1, PDE6B, ECE1, PRR3, CNOT6L, ENTPD7, ENTPD8, ZNF423, OBSCN, VAV3, SMG6, ITGA3, CPS1, VAV2, FURIN, CACNA2D2, FOXP1, CADPS2, EBF1, HIVEP3, CACNA1H, CACNA1C, CACNA1D, MYLK, CACNA1A, ATP7B, PC, CBFA2T3, ZNF205, ZKSCAN4, PDCD2, TRIM2, ZFYVE16, E4F1, TNIP2, CDK15, TERT, RTEL1, NDUFS1, NRXN2, ZNF280D, PFKL, NRXN3, ZNF91, RUNX1T1, ADIPOR1, NEK10, NRXN1, ZNF718, PRKD2, FOLH1, ACVR2B, EYA2, ZFYVE28, PRNP, PRKCZ, TIPARP, PPM1A, PRDM16, GLRX2, NGLY1, ZNF706, DDHD2, DDHD1, STK32B, PRDM15, STK32A, ZNF709, FCN2, ACAN, TGM6, ZNF606, GNAO1, RAF1, CREB5, COL5A1, CSGALNACT1, SP1, SUMF1, BMPR1B | 0.0003 | 1.2320 | 0.5698 |
| #7 | GO:0004114~3',5'-cyclic-nucleotide phosphodiesterase activity | 9 | 0.4978 | PDE6B, PDE1C, PDE4A, PDE4B, PDE1A, PDE5A, PDE10A, PDE4C, PDE6G | 0.0007 | 4.2810 | 1.0924 |
| #8 | GO:0031625~ubiquitin protein ligase binding | 44 | 2.4336 | TRAF1, FHIT, TRIOBP, USP2, GRIK2, RRAGA, RANGAP1, AURKA, BAG5, FBXW7, KBTBD4, BTBD1, PACRG, ASB10, BTBD3, TRAF6, SCN5A, AXIN1, RNF31, TRAF3, SPOP, EGFR, LTBR, SMG5, TMBIM6, TP53, UBE2F, SMAD3, RB1, UBE2L3, TANK, BTBD9, FZD6, RNF8, ATXN3, CUL4A, ARRB2, ARRB1, PRKAR1A, PIAS2, TNK2, USP25, UBE2U, BTBD11 | 0.0008 | 1.6773 | 1.3638 |
| #9 | GO:0005096~GTPase activator activity | 43 | 2.3783 | ARFGAP1, RAP1GAP, ASAP1, MYO9B, RANGAP1, ARHGAP17, TBC1D19, NPRL3, LLGL2, TBC1D16, RASAL1, TBC1D15, ARHGAP22, DOCK1, RGS12, TBC1D4, RANBP1, AGAP1, RAP1GAP2, PLCB1, AGAP3, AXIN1, ELMOD1, DAB2IP, VAV3, BCR, ABR, PLXNB1, NF1, ECT2, TBC1D22A, TBCK, ARHGAP26, RGS20, ARRB1, SIPA1L1, TSC2, GRTP1, CHN1, RGS6, SRGAP3, CHN2, KALRN | 0.0009 | 1.6862 | 1.4144 |
| #10 | GO:0004713~protein tyrosine kinase activity | 25 | 1.3827 | EGFR, FGF5, FGFR4, BCR, FGFR3, FGF9, NEK1, AXL, FER, FES, TTN, EPHB2, IGF1R, PTK2, EPHA6, CLK3, PTK2B, DYRK1A, ZAP70, TNK2, CUX1, FGF1, NRG2, INSR, MAP2K5 | 0.0009 | 2.0565 | 1.4919 |
